# A Novel Approach for Position Verification and Dose Calculation through Local MVCT Reconstruction

**DOI:** 10.3390/diagnostics14050482

**Published:** 2024-02-23

**Authors:** Jun Zhang, Zerui Chen, Yuxin Lei, Junhai Wen

**Affiliations:** 1 College of Computer Science and Technology, Taiyuan University of Technology, Taiyuan 030024, China; zhangjun08@tyut.edu.cn; 2 School of Life Science, Beijing Institute of Technology, Beijing 100081, China; 3220231967@bit.edu.cn (Z.C.); leiyx@bit.edu.cn (Y.L.)

**Keywords:** EPID, MVCT, dose calculation, positioning verification, registration

## Abstract

Traditional positioning verification using cone-beam computed tomography (CBCT) may incur errors due to potential misalignments between the isocenter of CBCT and the treatment beams in radiotherapy. This study introduces an innovative method for verifying patient positioning in radiotherapy. Initially, the transmission images from an electronic portal imaging device (EPID) are acquired from 10 distinct angles. Utilizing the ART-TV algorithm, a sparse reconstruction of local megavoltage computed tomography (MVCT) is performed. Subsequently, this MVCT is aligned with the planning CT via a three-dimensional mutual information registration technique, pinpointing any patient-positioning discrepancies and facilitating corrective adjustments to the treatment setup. Notably, this approach employs the same radiation source as used in treatment to obtain three-dimensional images, thereby circumventing errors stemming from misalignment between the isocenter of CBCT and the accelerator. The registration process requires only 10 EPID images, and the dose absorbed during this process is included in the total dose calculation. The results show that our method’s reconstructed MVCT images fulfill the requirements for registration, and the registration algorithm accurately detects positioning errors, thus allowing for adjustments in the patient’s treatment position and precise calculation of the absorbed dose.

## 1. Introduction

Radiation therapy is a common cancer treatment method emphasizing precise irradiation of tumor areas and minimizing damage to normal surrounding tissue, which is a research focus in the field of radiation therapy [1,2,3,4]. Intensity-modulated radiation therapy (IMRT) and volumetric-modulated arc therapy (VMAT) represent two advanced radiotherapy techniques that adjust radiation dose distribution to target tumor areas precisely while minimizing damage to healthy surrounding tissue. These two techniques have significantly improved treatment precision, reduced side effects, and enhanced patients’ quality of life [5]. IMRT and VMAT technologies have offered greater flexibility in radiation therapy, but have also introduced complexities in treatment planning and dose delivery [6]. Errors during treatment can lead to deviations in dose distribution, affecting the effectiveness of patient treatment.

In radiation therapy, accurate patient positioning is crucial to ensure precise dose delivery and minimize damage to normal surrounding tissue, thereby reducing the risk of side effects and complications [4,7,8,9]. Positioning verification, a critical step in the treatment process, aims to ensure that patients consistently align with the planned position during treatment, ensuring accurate radiation dose delivery to the target area. In recent years, imaging-guided techniques such as cone-beam CT (CBCT) and digitally reconstructed radiographs (DRRs) have been widely used in positioning verification for radiation therapy [10,11]. DRR images are typically reconstructed from planned CT scans for two-dimensional plane registration. Simultaneously, CBCT can provide three-dimensional patient images, offering a more accurate depiction of patient anatomy and aiding in precise positioning verification. According to a national survey conducted by the American Society for Radiation Oncology, the utilization rate of CBCT in image-guided radiation therapy (IGRT) increased from zero in the early 21st century to 92% in 2015 [12]. However, using CBCT for positioning verification also presents some drawbacks. CBCT is typically integrated into the accelerator gantry using a separate radiation source, and its use may result in discrepancies between its rotation center and the treatment isocenter of the accelerator. Like any linear accelerator accessory device used for patient positioning, it must undergo strict spatial accuracy testing, among which the isocenter consistency test is critical [13,14,15,16]. Moreover, CBCT is not part of the basic configuration of accelerators and usually requires additional procurement, increasing the burden on hospitals. Additionally, traditional position verification techniques, such as cone-beam computed tomography (CBCT), apply additional radiation doses to patients, which may lead to complications in normal surrounding tissue [17,18].

The electronic portal imaging device (EPID) is a commonly used image acquisition device integrated into the accelerator and can be used without cumbersome positioning procedures. Its features include excellent image display, a large imaging area, and high radiation resistance. Initially used for two-dimensional imaging-based positioning verification of patients, the EPID’s excellent dose linearity response has extended its application to patient dosimetry verification. Van Elmpt et al. [19] employed EPID transmission images combined with the patient’s CBCT to calculate the accelerator’s output fluence distribution inversely. Then, using Monte Carlo algorithms, they calculated the dose distribution within the patient’s CBCT, validating it against doses computed by treatment planning systems (TPSs). Van Uytven et al. [20] utilized an iterative approach to compute the accelerator’s output fluence distribution from EPID transmission images. Subsequently, it calculated the dose distribution within the patient’s planned CT and validated it against TPS-calculated values. Wendling et al. [21,22,23] calculated primary beam dose values within various phantom layers by computing the transmission rates of both EPID open-field and EPID transmission images. After scatter correction, they further computed the dose distribution within the phantom, comparing it against TPS-planned values. Pecharroman-Gallego et al. [24] modeled the transmission rates for various fields, calculating doses at the central plane of the phantom after correcting for softening and hardening effects of radiation beams, avoiding the need for repeated execution of treatment plans to measure open-field images. Currently, most research focuses on using EPIDs to reconstruct the three-dimensional dose distribution of patients or phantoms. When using EPIDs for dose verification, it is crucial to ensure consistency between the patient’s positioning information and the planned positioning in the treatment design, making pre-treatment positioning verification pivotal. Due to the similar megavoltage level radiation source used during EPID image acquisition and treatment, the tissue contrast in EPID transmission images is relatively poor, leading to limited research on three-dimensional image reconstruction and positioning verification using EPIDs.

Our team conducted earlier research on pre-treatment dose verification based on EPID [25] and in vivo dose verification methods [25,26], discovering that EPID transmission images contain patient anatomical information, which can be utilized for reconstructing the three-dimensional structure of patients. Furthermore, the radiation source used for EPID image acquisition is the same as that used during patient treatment, which addresses the limitations of CBCT for positioning verification to some extent. Therefore, based on our earlier research, this paper proposes a radiation therapy positioning verification and dose calculation method based on localized megavoltage computed tomography (MVCT) reconstruction. By utilizing EPID two-dimensional transmission images, we reconstruct localized structural information of the patient (i.e., MVCT) and then register the reconstructed MVCT with planned CT for positioning verification. Subsequently, adjustments can be made to the patient’s treatment position, enabling precise calculation of absorbed patient doses.

## 2. Materials and Methods

### 2.1. Materials

During the measurement process, the accelerator utilized was the Varian Halcyon Linac (Varian Medical Systems, Palo Alto, CA, USA). All measurements were performed using 6 MV X-rays, with a dose rate of 800 mu/min. The EPID detector used was the aS1200, featuring an effective measurement area of 43 cm × 43 cm, with a corresponding detector count of 1280 × 1280. The distance between the EPID and the accelerator source was 1540 mm. The phantom employed was the CIRS002LFC thorax phantom (CIRS, Norfolk, VA, USA).

### 2.2. Algorithm Description

**Figure 1:**
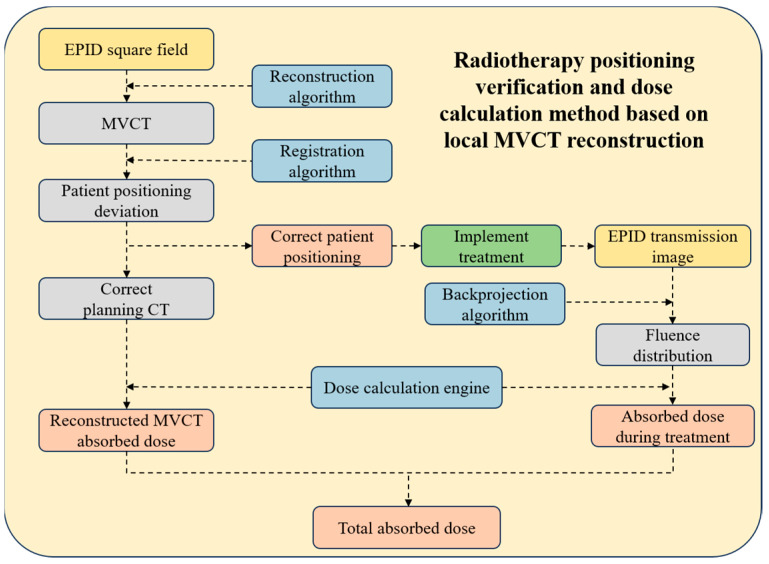
The flowchart of our model.

This paper proposes a novel method for radiation therapy positioning verification and dose calculation based on localized MVCT reconstruction. The method initially involves reconstructing the patient’s three-dimensional anatomical information by collecting EPID transmission images. Subsequently, a three-dimensional registration algorithm aligns the reconstructed three-dimensional image with the planned CT scan, enabling the determination of the patient’s positioning error. Based on this positional error information, adjustments are made to the patient’s treatment position. The method incorporates the output fluence during treatment into the dose calculation engine to compute the actual dose received by the patient. Additionally, the dose absorbed by the patient during the registration process is integrated into the total absorbed dose in each part of the patient’s anatomy. The specific workflow is illustrated in Figure 1.

### 2.3. MVCT Reconstruction

**Figure 2:**
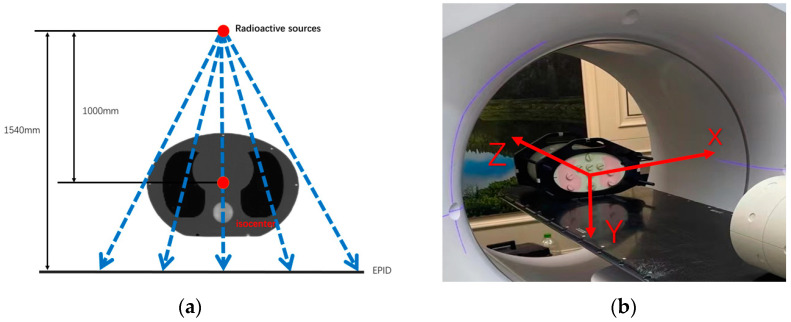
(**a**) Schematic representation of the phantom positioning. (**b**) Three-dimensional coordinate diagram.

The MVCT reconstruction process was validated using a CIRS thorax phantom, with a distance of SAD = 1000 mm between the phantom center and the accelerator source, as depicted in Figure 2a. The corresponding three-dimensional coordinates are illustrated in Figure 2b.

**Figure 3:**
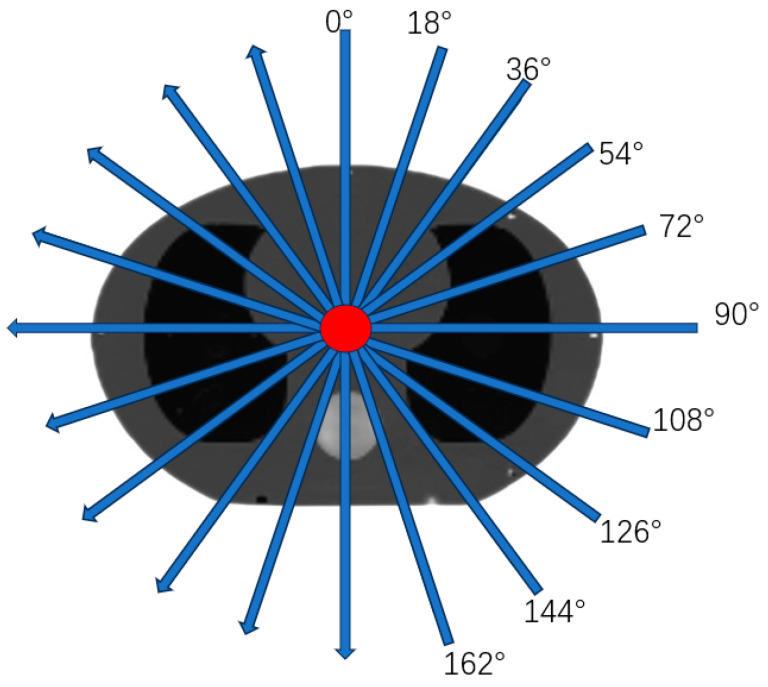
Schematic diagram of irradiation angle.

The accelerator’s monitor unit is configured as 1 mu, and the field size is set to 20 cm × 20 cm. Ten angles of EPID open-field images and transmission images are acquired within the range of 0–180 degrees (spaced at intervals of 18 degrees) on the gantry (as shown in Figure 3). The image obtained for phantom positioning is KVCT.

MVCT reconstruction employs a back-projection reconstruction algorithm, initially used in CT reconstruction. During measurement, the detector collects X-ray intensity values after passing through tissue attenuation. Combining these attenuated intensity values with their initial intensity before attenuation allows the calculation of projection values. An approximate reconstructed image can be obtained by back-projecting these projection values from different angles into the region of the reconstructed object. In this paper, the EPID open-field images are collected as initial X-ray intensity values, and the EPID transmission images represent the intensity values of X-rays passing through patient tissue after attenuation. Subsequently, Formula (1) is utilized to convert the obtained EPID open-field images and transmission images into projection images:

(1) $$P=ln(I_{0}/I)$$

$P={\left(p_{1},p_{2},\cdot \cdot \cdot p_{i}\right)}^{T}$ represents the projection image, $p_{i}$ denotes the projection value of the *i*th ray, $I_{0}$ is the grayscale value of the EPID open-field image, and I stands for the grayscale value of the EPID transmission image when the patient is present.

To accelerate the speed of MVCT image reconstruction, preprocessing of the projection images is necessary to reduce complexity. The original images undergo the following processing steps.

The bilinear interpolation method is employed to resample the images to balance computational efficiency and image quality. This not only enhances the algorithm’s overall performance with a relatively small computational cost but also helps avoid excessive smoothing or loss of details in the image, ensuring the preservation of the overall structure of the original image. Using bilinear interpolation, the pixel size of the image is adjusted from the original 0.336 mm × 0.336 mm to 1 mm × 1 mm, resulting in an image size of 300 × 400 pixels.

Since the treatment field area generally does not exceed the size of the detection panel, there are invalid regions, represented by pixel values of 0, in the projection images. Therefore, the ineffective areas at the edges are cropped, reducing the image size to 300 × 300 pixels.

The images are resampled again, adjusting the pixel size to 3 mm × 3 mm. The final size of the projection image is 100 × 100 pixels. These preprocessing steps enhance algorithm efficiency while ensuring the reconstructed image maintains good quality and structural integrity.

**Figure 4:**
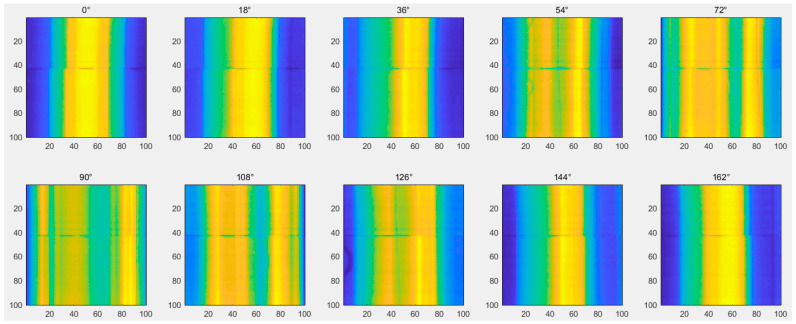
The preprocessed projection images.

Figure 4 displays a set of preprocessed projection images obtained in an experiment.

**Figure 5:**
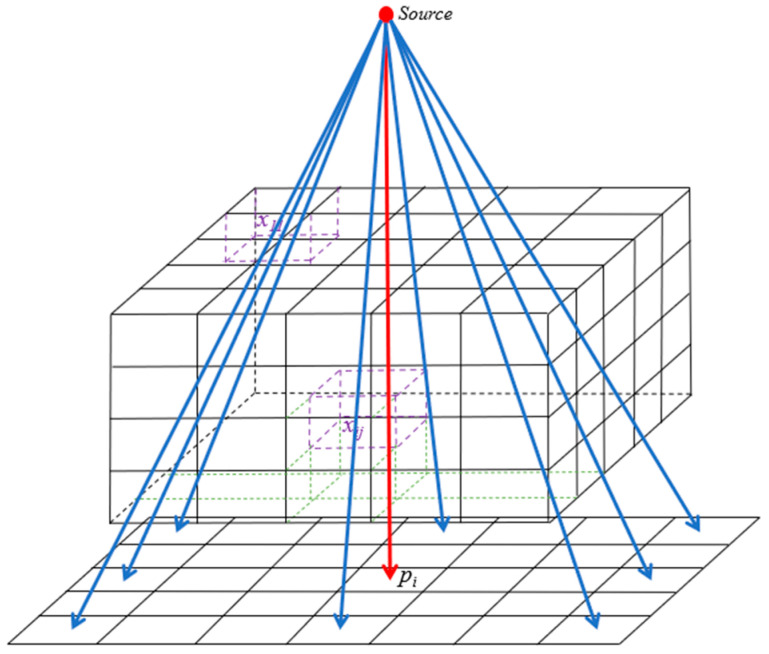
The reconstruction model.

After acquiring the projection images, this study utilized the algebraic reconstruction technique (ART) to reconstruct the patient’s MVCT images. The fundamental principle of ART involves starting with an initial image $x^{\left(0\right)}$, performing projection operations, comparing the resultant outcome with the measured actual projection data, and subsequently adjusting the initial image based on the differences to obtain the next iterative image $x^{\left(1\right)}$. This process continues through successive adjustments. The ART algorithm calculates each ray individually within the field of view. Upon completion of calculations for each ray, the image is updated once. After completing calculations for all rays, one algorithm iteration is finished. The formula for the algorithm is as follows:

$$x^{\left(0\right)}=Any\;initial\;value$$

(2) $$x^{\left(n+1\right)}=\left\{\begin{array}{c} x^{\left(n\right)},\;\;\;\;\;\;\;\;\;\;\;\;\;\;\;\;\;\;\;\;w_{i}^{T}x^{\left(n\right)}\leq p_{i}\\ x^{\left(n\right)}+\lambda^{\left(n\right)}\frac{p_{i}-w_{i}^{T}x^{\left(n\right)}}{{\left\|w_{i}\right\|}^{2}}w_{i},\;other\;\; \end{array}\right.$$

where $x^{\left(0\right)}$ represents the initial image, $x^{\left(n+1\right)}$ represents the corrected image, i represents the *i*-th ray, p represents the measured projection data, w is the projection coefficient matrix, $w_{i}\;$ represents the projection coefficient of the *i*-th ray, and λ is the relaxation factor. Figure 5 depicts the reconstruction model.

We incorporated the total variation (TV) regularization strategy into the reconstruction model to achieve better image reconstruction quality. Regularization can constrain the reconstruction process, and a typical regularization method is TV regularization. The TV norm is the L1 norm of the gradient. The TV of image x can be defined as shown in Equation (3):

(3) $${\left\|x\right\|}_{TV}=\sum_{i,j}\left|\vec{\nabla }x_{i,j}\right|=\sum_{i,j}\sqrt{{\left(x_{i,j}-x_{i-1,j}\right)}^{2}+{\left(x_{i,j}-x_{i,j-1}\right)}^{2}}$$

where $x_{i,j}$ represents the pixel value of the *i*-th row and *j*-th column in the image.

In our reconstruction algorithm, the purpose of minimizing the TV norm of the image is to make the image as flat as possible. TV regularization involves solving for the minimum TV norm of the image, aiming to reduce the gradient values to zero across as many regions as possible, thereby rendering the image as smooth as possible. In this scenario, the gradient descent algorithm can be employed. The formula for calculating the image gradient is shown in Equation (4):

(4) $$\begin{array}{ll} v_{i,j}=\frac{\partial {\left\|x\right\|}_{TV}}{\partial x_{i,j}} & \approx \frac{\left(x_{i,j}-x_{i-1,j}\right)+\left(x_{i,j}-x_{i,j-1}\right)}{\sqrt{{\left(x_{i,j}-x_{i-1,j}\right)}^{2}+{\left(x_{i,j}-x_{i,j-1}\right)}^{2}+\varepsilon }}\\ & -\frac{\left(x_{i+1,j}-x_{i,j}\right)}{\sqrt{{\left(x_{i+1,j}-x_{i,j}\right)}^{2}+{\left(x_{i+1,j}-x_{i+1,j-1}\right)}^{2}+\varepsilon }}\\ & -\frac{\left(x_{i+1,j}-x_{i,j}\right)}{\sqrt{{\left(x_{i,j+1}-x_{i-1,j+1}\right)}^{2}+{\left(x_{i,j+1}-x_{i,j}\right)}^{2}+\varepsilon }} \end{array}$$

During the computation, to prevent the image’s TV norm from yielding an infinite value when calculated, a very small parameter ε is introduced in the denominator to avoid the value approaching infinity as ε tends towards zero.

The reconstruction of the MVCT image utilizes the ART-TV algorithm. During the reconstruction process, the number of iterations in ART positively correlates with improving iteration effects. Experiments have shown that after 12 iterations, changes in the reconstructed image approach zero, making the image suitable for registration. The selection of the ART relaxation factor should be appropriate, as an optimal relaxation factor can enhance both the speed and quality of reconstruction. Generally, choosing a relaxation factor between 0.05 and 0.25 yields better reconstruction results. The TV regularization parameter significantly affects image smoothing: a larger parameter accelerates the convergence of the reconstructed image and increases smoothness. However, overly large parameters may lead to non-convergence and excessive smoothing of the reconstructed image. The specific iterations are as follows.
(1)Required parameters for initialization of reconstruction: maximum number of iterations, the relaxation factor, TV regularization parameters, the initial image $x^{\left(0,0\right)}.$(2)In the *t*-th iteration, for the *k*-th ray:
➀Process using the ART algorithm of Equation (2) to obtain the updated image $x^{\left(t,k\right)}$.➁Apply non-negative constraints on $x^{\left(t,k\right)}\;$ to obtain $x_{pos}^{\left(t,k\right)}$; non-negative constraints are as follows:
$$x_{pos}^{\left(t,k\right)}=\left\{\begin{array}{c} x^{\left(t,k\right)},\;x^{\left(t,k\right)}>0\\ 0,\;\;\;\;x^{\left(t,k\right)}<0 \end{array}\right.$$
➂Calculate difference measurement d$,\;d={\left\|x^{\left(t,k-1\right)}-x_{pos}^{\left(t,k\right)}\right\|}_{2}$.➃Use $x_{TV}^{\left(t,k\right)}=x_{pos}^{\left(t,k\right)}-\mu \mathrm{d}\frac{v}{{\left\|v\right\|}_{2}}$ to minimize TV and obtain $x_{TV}^{\left(t,k\right)}$, where μ  is the regularization parameter of TV, and v is calculated using Formula (4).➄Make $x^{\left(t,k-1\right)}=x_{TV}^{\left(t.k\right)}$ and execute the next ray.➅After all rays have been executed, make $x^{\left(t-1\right)}=x_{TV}^{\left(t\right)}\;$ and enter the next iteration.(3)Repeat step (2) until the maximum number of iterations is reached or the difference measurement d reaches a minimal value.

### 2.4. Image Registration Algorithm

The reconstructed MVCT reflects the actual pre-treatment positioning information of the patient, and comparing it with the planned CT allows us to determine the patient’s positional deviation. Our algorithm utilizes mutual information to register MVCT and planned CT. Mutual information is a similarity measure based on Shannon’s entropy, used to quantify the correlation between systems.

The mutual information between two systems can be described using the concept of entropy as follows:

(5) MI(A,B)=H(A)+H(B)−H(A,B)

where H(A) and H(B) represent the entropy of systems A and B respectively, and H(A,B) is their joint entropy.

Shannon’s entropy measures the amount of information contained in a message and the uncertainty of events. Assuming a random phenomenon has n possible outcomes, each occurring with probabilities $p_{1},p_{2},\cdots ,p_{n}$, then the Shannon entropy is defined as:

(6) $$H=\sum_{i}p_{i}\log_{2}\frac{1}{p_{i}}=-\sum_{i}p_{i}\log_{2}p_{i}$$

The various entropies in Equation (5) can be represented as follows:

(7) $$H(A)=-\sum_{a}p_{A}(a)\log_{2}p_{A}(a)$$

(8) $$H\left(B\right)=-\sum_{b}p_{B}(b)\log_{2}p_{B}(b)$$

(9) $$H(A,B)=-\sum_{a,b}p_{AB}(a,b)\log_{2}p_{AB}(a,b)$$

where $p_{A}(a)$ and $p_{B}(b)$ represent the probability distributions of systems A and B, respectively, and $p_{AB}(a,b)$ represents the joint probability distribution of both systems.

In this paper, we performed three-dimensional rigid registration between the localized MVCT and the planned CT to determine the patient’s positional error. Considering the localized MVCT as the floating image denoted by R and the planned CT as the reference image characterized by F, we represent their mutual information in terms of entropy as shown in Equation (10):

(10) $$MI(R,F)=-\sum_{r}p_{R}(r)\log_{2}p_{R}(r)-\sum_{f}p_{F}(f)\log_{2}p_{F}(f)+\sum_{r,f}p_{RF}(r,f)\log_{2}p_{RF}(r,f)$$

In this equation, $P_{R}\left(r\right)$ and $P_{F}\left(r\right)$ represent the grayscale probability distributions of the floating image R and the reference image F, respectively, while $P_{RF}\left(r,f\right)$ denotes the joint probability distribution of these two images.

**Figure 6:**
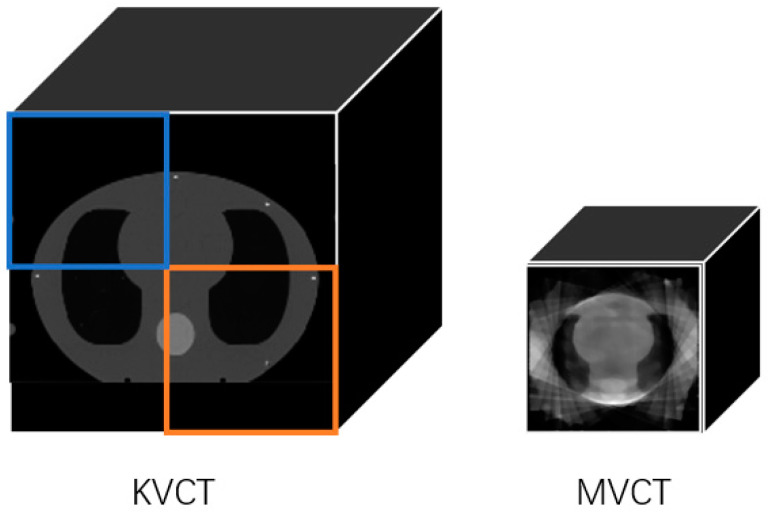
Registration process. Local MVCT is the reference window, while the planning CT is the search area.

Figure 6 depicts the registration process, where the local MVCT serves as the reference window and the planning CT functions as the search area. The coordinates of the planning CT’s center, set in the treatment plan, are $\left(x_{0},y_{0},z_{0}\right)$. The reference window slides with a one-pixel step from the top left to the bottom right within the search area. After each movement, the mutual information (MI) value of the respective positions in both images is calculated. The coordinates $\left(x,y,z\right)$, corresponding to the maximum MI value, determine the center point representing the patient’s pre-treatment positioning. The difference between these coordinates, calculated according to Equation (11), represents the patient’s positioning error, denoted as t.

(11) $$t=\left(t_{x},t_{y},t_{z}\right)=(\left(x_{0}-x\right)*{pixelsize}_{x},\left(y_{0}-y\right)*{pixelsize}_{y},\left(z_{0}-z\right)*{pixelsize}_{z})$$

Here, ${pixelsize}_{x}$, ${pixelsize}_{y}$ and ${pixelsize}_{z}$ represent the actual dimensions of the pixels in the *x*, *y*, and *z* directions of the MVCT and planning CT, respectively.

### 2.5. Back-Projection Algorithm

**Figure 7:**
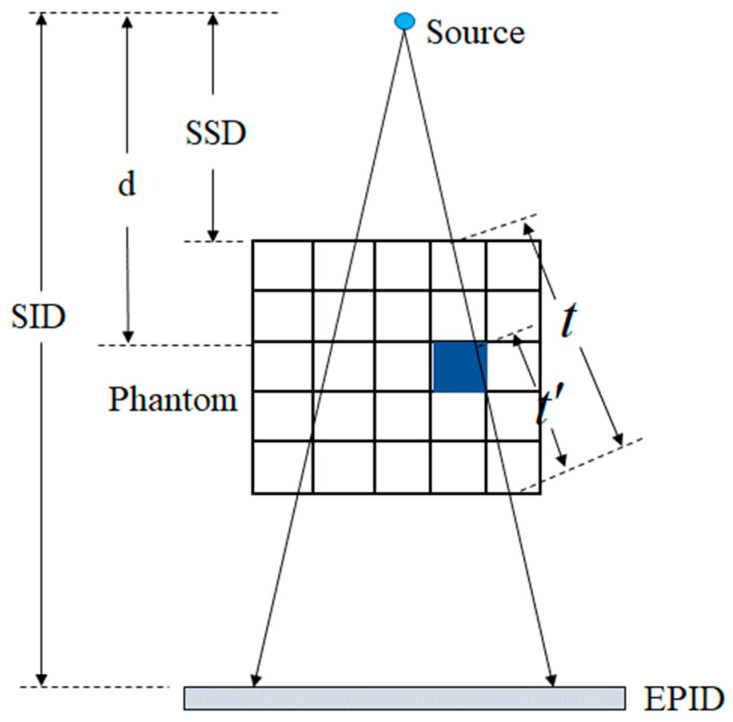
Parameter diagram of back-projection algorithm model.

The back-projection algorithm can calculate the intensity values of X-rays before incident on the patient through the EPID transmission image, that is, the output intensity distribution of the accelerator. Once corrections are made for patient-positioning deviations, treatment can be administered. During the treatment process, the EPID transmission images are collected. Based on our previously developed EPID dose verification algorithm [27], after scatter correction, attenuation correction, and grayscale-intensity conversion, the EPID transmission images can be utilized to calculate the primary fluence values of the accelerator, as shown in Equation (12):

(12) $$\psi^{p}\left(t^{\prime },r,d\right)=f\cdot \frac{{SID}^{2}}{d^{2}}\cdot \psi_{EPID}^{p}(t,r)/exp(-\frac{a(r)\cdot t^{\prime }}{1+b(r)\cdot t^{\prime }})$$

where $\psi^{p}(t^{\prime },r,d)$ represents the primary fluence of the accelerator’s rays. Because the unit of the EPID image is a grayscale value, which is different from the unit of accelerator fluence value, it needs to be converted through a conversion coefficient f, which signifies the linear conversion parameter of the EPID image’s grayscale value to the accelerator’s intensity value. SID stands for the distance from the accelerator source to the EPID plane, d denotes the perpendicular distance from the accelerator source to the calculation point within the body, $t^{\prime }$ accounts for the equivalent thickness from the EPID plane to the calculation point, t is the equivalent thickness along the path from the accelerator source to the calculation point on the EPID plane, $\psi_{EPID}^{p}(t,r)$ represents the primary value of the rays at the EPID plane, and a(r) and b(r) represent the attenuation coefficients of the rays. With the increasing off-axis distance, X-rays will undergo a softening effect. In addition, the beam hardening can be caused by the patient/phantom placed in the beam. We use a(r) and b(r) to correct the softening effect and hardening effect of the rays, respectively. The schematic is illustrated in Figure 7.

After calculating the accelerator’s emitted intensity for each field, the obtained intensity values can be used as inputs for any dose calculation engine algorithm to calculate the actual dose received by the patient’s body, such as the pencil beam algorithm (Equation (13)) [28]:

(13) $$D\prime (x,y,d)=\sum_{j=1}^{3}{D_{j}}^{\prime }(d)\cdot [\Psi^{p}⊗W_{j}](x,y)$$

where $\Psi^{p}\;$ denotes the intensity of the accelerator’s emission, ⊗ represents convolution, ${D_{1}}^{\prime }(d)$ stands for the dose generated by primary radiation, ${D_{2}}^{\prime }(d)$ signifies the dose caused by small-scale scatter, ${D_{3}}^{\prime }(d)$ indicates the dose generated by large-scale scatter, and $W_{i}(x,y)$ denotes the weighting of the corresponding components in the deposition kernel.

We used the EPID transmission images to reconstruct the local MVCT, enabling the computation of the absorbed dose during reconstruction. It is essential to note that when calculating the absorbed dose during the MVCT reconstruction, it is imperative to initially correct the planned CT position based on the registration results to align it with the actual patient position. Subsequently, Formulas (12) and (13) are employed to calculate the actual absorbed dose distribution by the patient during the acquisition of the ten angle EPID projection images. The cumulative dose absorbed by the patient during the reconstruction of the MVCT and the dose received during the treatment process constitute the total dose received in that treatment session. This total dose could serve as a basis for optimizing subsequent treatment plans.

## 3. Results

### 3.1. MVCT Reconstruction Results

**Figure 8:**
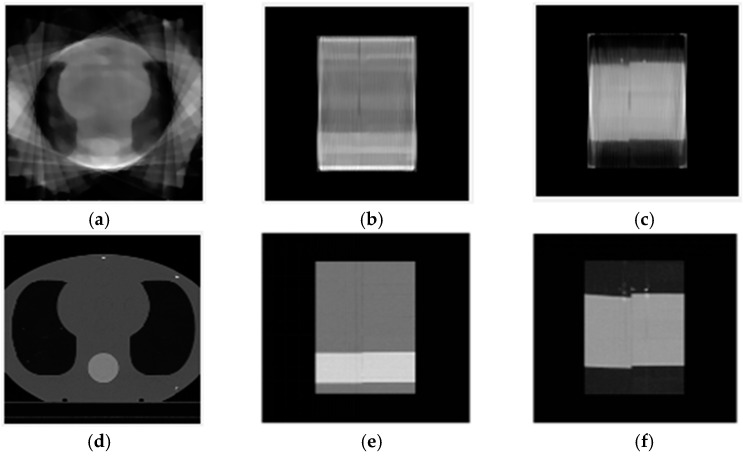
(**a**) Cross-sectional view of the MVCT reconstruction result, (**b**) sagittal view of the MVCT reconstruction result, (**c**) coronal view of the MVCT reconstruction result, (**d**) cross-sectional view of planned CT, (**e**) sagittal view of planned CT, and (**f**) coronal view of planned CT.

The MVCT image reconstruction was performed using the ART-TV algorithm on the pre-processed projection data. The reconstructed image size was 128 × 128 × 122 pixels, with each pixel measuring 2 × 2 × 3 mm^3^. The maximum number of iterations for ART is set to twelve, the relaxation factor is set to 0.1, and the TV regularization parameter is set to 1, accelerating the iterative reconstruction process. As shown in Figure 8, the reconstructed MVCT is compared with the planning CT, which has a size of 256 × 256 × 122 pixels, with each pixel measuring 2 × 2 × 3 mm^2^. The cross-sectional view represents the 61st layer of the three-dimensional image, while the sagittal and coronal views represent 64 layers each.

### 3.2. Registration Result

When validating the image registration results, we first positioned the patient to ensure that the central point’s location matched the planned treatment center. While maintaining the patient’s position on the treatment bed unchanged, we simulated the positioning deviation during treatment by manually adjusting the treatment bed’s position using the console. The simulation parameters are outlined in Table 1.

**Figure 9:**
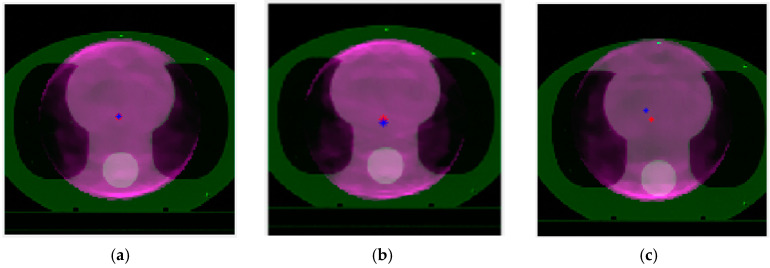
(**a**) Registration results without any movement from the initial position, (**b**) alignment after a 3 mm upward movement, and (**c**) after shifting 10 mm downwards and 5 mm to the right. The red dots represent the center point of the KVCT, while the blue dots indicate the registered center points.

After adjusting the positioning deviation, 10 EPID transmission images were captured at different angles. The reconstruction algorithm from Section 2.3 was used to reconstruct the MVCT image of the phantom, followed by alignment using the registration algorithm from Section 2.4. The aligned MVCT and the corresponding region of the planned CT were superimposed for display. Figure 9 illustrates the alignment results of the transverse sections related to the first, third, and fourth groups in Table 1, where Figure 9a represents the alignment results of the first group, Figure 9b for the third group, and Figure 9c for the fourth group. The red dots indicate the center point of the planned CT, while the blue dots represent the registered center point.

Table 2 presents the results of registering the simulated positioning deviations from Table 1. The actual treatment center points of the phantom in the *x* and *y* directions in the planned CT are (128.5, 126.5). The artificially moved treatment bed position serves as the reference to validate the registration results. Upon comparison, it was observed that the registration error for the first simulated positioning deviation was 0 mm, while for the other simulated deviation, the registration error was 1 mm, falling within clinically acceptable limits.

### 3.3. Results of the Back-Projection Algorithm

**Figure 10:**
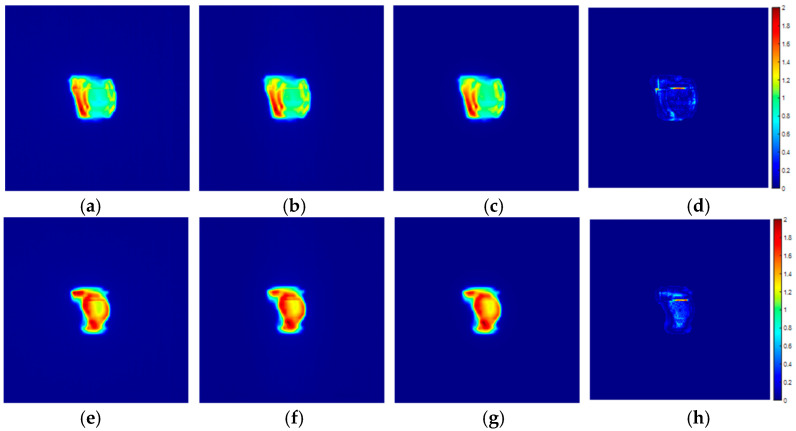
IMRT inversion calculation results. (**a**,**e**) EPID transmission images, (**b**,**f**) calculated accelerator output intensity from the back-projection algorithm, (**c**,**g**) accelerator output intensity measured in the open field, and (**d**,**h**) gamma distribution between the inversion-calculated and open-field measured output intensities.

To validate the accuracy of the inversion algorithm, we collected EPID open-field images as reference values. Then, using a linear conversion factor, we transformed the EPID open-field image values into the accelerator’s output fluence values. For validation, we replicated the actual treatment plan with the CIRS phantom. The EPID transmission images collected during treatment were used with the back-projection algorithm to compute the accelerator’s output fluence values. The fluence obtained from the converted open-field image was used as the reference value to calculate the global gamma passing rate (3%/2 mm criteria, 10% threshold), which was consistently greater than 95%. Figure 10 illustrates the results of two different IMRT field calculations.

## 4. Discussion

In this study, we developed a novel method for verifying patient positioning in radiotherapy. Before patient treatment begins, EPID transmission images from 10 different angles are collected to reconstruct the patient’s MVCT. This MVCT is then registered with the planning CT to verify the patient’s positioning errors. The patient’s treatment position is adjusted based on the registration results before proceeding. After treatment, the back-projection algorithm calculates the fluence value of the accelerator during reconstructed MVCT and treatment. The fluence values are input into a dose calculation engine to compute the total dose absorbed by the patient, which can then be used to optimize subsequent treatment plans. Our study aimed to enhance the speed and efficiency of MVCT reconstruction. The collected EPID transmission images were initially preprocessed and down-sampled to reduce their resolution. Subsequently, the ART-TV algorithm was employed to reconstruct and process the EPID projection images. The entire reconstruction procedure required collecting only 10 angular data points, thus reducing radiation dose while ensuring favorable reconstruction outcomes. The registration results indicate that the use of MVCT images reconstructed from 10 angles meets the registration requirements, with the accuracy falling within clinically acceptable ranges.

Relevant studies indicate that the accuracy of using CBCT for radiotherapy setup verification is influenced by several factors, including tumor type, patient positioning, and imaging frequency, with an average error of approximately 1 mm [29]. In our study, verification was conducted using a CIRS thorax phantom, with an error of 1 mm, which is comparable to the accuracy achieved when using CBCT for positioning verification. However, in contrast to CBCT, direct utilization of the same megavoltage radiation source as employed during therapy for reconstruction and registration obviates the necessity of configuring two different energy sources within a single accelerator. This contributes to reducing the complexity of accelerator configurations to some extent and avoids positioning errors resulting from inconsistencies between the rotation center of the CBCT device and the accelerator center. Consequently, this streamlines operational procedures and decreases medical costs. Our reconstruction model solely collected projection data within the 0- to 180-degree range, from which 10 angular data points were obtained, diminishing the intensity of X-rays emitted from the machine.

Furthermore, any additional dose absorbed by the patient during the registration process can be included in the total absorbed dose across various phantom sections. This enables accurate computation of the total dose absorbed by the patient, serving as a basis for optimizing subsequent patient treatment plans.

The registration algorithm compares and analyzes the quantitative results obtained from the registration outcomes against the defined “gold standard” (the displacement parameters set during data collection). Table 2 demonstrates that certain registration errors exist in groups 3, 4, and 5. These errors primarily stem from the search process, where this study searched with a one-pixel step, meaning a step of 2 mm in the *x* and *y* directions and 3 mm in the *z* direction. When the registration error is less than 2 mm in the *x* and *y* directions, it can be considered a systematic error caused by the precision of pixels.

During the registration validation process, we simulated displacement deviations only in the *x* and *y* directions and did not validate deviations in the *z* direction. This was mainly due to limitations in our experimental conditions. The CIRS phantom used for validation currently does not offer similar organizational structures in the *z* direction (as shown in Figure 8), which does not meet the conditions required for registration. In the future, we will continue to select other phantoms and actual patients to verify the accuracy of our method.

During the patient treatment process, if the dose distribution received by the patient can be monitored at the end of each treatment, it can be used to adjust and optimize subsequent treatment plans according to the actual situation to improve the quality of patient treatment. The EPID is always positioned at the end of the radiation beam during patient treatment and does not affect the radiation fluence distribution inside the patient’s body. The transmission images collected by the EPID are closely related to the patient’s anatomical structure, making the EPID an ideal device for monitoring the fluence intensity during the patient treatment process. The grayscale values of the EPID transmission images are proportional to the accelerator’s emission intensity, and their original radiation grayscale contribution values follow exponential decay laws and inverse square laws. These images can be used to compute the fluence distribution of X-rays passing through the pathway directly during the treatment process, which is then used to calculate the actual dose received by the patient. Due to significant differences in dose calculation algorithms for correcting non-uniform tissue, we have not yet implemented a commercial dose calculation algorithm. Therefore, in validating the accuracy of the back-projection algorithm, we did not use the dose distribution within the phantom as a comparison result. Instead, we used the accuracy of the accelerator’s fluence calculated through back-projection algorithm calculation as an evaluation criterion. If the accelerator’s fluence calculated through the back-projection algorithm from EPID transmission images matches the actual accelerator’s fluence value, then the dose distribution within the patient’s body calculated using the same dose calculation algorithm will also be identical. The acquisition method of EPID transmission images during the back-projection algorithm calculation process is crucial. The back-projection algorithm requires the separate acquisition of EPID transmission images for each angle in the treatment plan for inversion. Due to the hardware limitations of the accelerator, the current mode we use for acquiring EPID images is an integrated mode. It is impossible for VMAT plans to capture EPID transmission images for each angle separately. Therefore, our back-projection algorithm is currently not suitable for VMAT plans.

There may be some limitations in this study. (1) Due to constraints in experimental conditions, our verification was conducted solely using a CIRS thorax phantom without using patient data. Owing to the insufficient heterogeneity of the CIRS phantom in the *Z*-axis, detection along the *Z*-axis was not performed. (2) The accuracy of the registration search is limited to one pixel. (3) We have not yet developed a commercial dose calculation algorithm; hence, dose calculation has not undergone end-to-end validation. (4) The long-term clinical efficacy also requires further refinement. We will address these shortcomings in future work.

## 5. Conclusions

This paper proposes a novel method for radiation therapy positioning verification and dose calculation based on local MVCT reconstruction. Initially, 10 angle-specific EPID transmission images are collected to reconstruct the patient’s local MVCT. Subsequently, a three-dimensional registration method is employed to register the reconstructed image with the planned CT, acquiring information on patient-positioning errors. Based on this information, adjustments are made to the patient’s treatment position. The method utilizes imaging from the radiation source during treatment, eliminating the need for configuring two different energy sources on the same accelerator. Moreover, it avoids positioning errors arising from discrepancies between the CBCT device’s rotation center and the accelerator’s center. During registration, the additional dose absorbed by the patient is incorporated into the overall absorbed dose of each body part. After each treatment session, subsequent treatment plans can be adjusted based on the total dose absorbed by the patient thus far. Experimental results demonstrate that our method’s MVCT image quality meets registration requirements. The mutual information algorithm accurately identifies positioning errors, enabling adjustments to the patient’s treatment position and precise calculation of the patient’s absorbed dose.

## Figures and Tables

**Table 1:** Simulated experimental data of displacement parameters.

Experiment Number	Displacement Parameters	
	X-Direction/mm	Y-Direction/mm
Group 1	0	0
Group 2	−10	0
Group 3	0	−3
Group 4	5	10
Group 5	3	−3

**Table 2:** Registration results and position error.

Experiment Number	Registration Results		Gold Standard	Registration Error (∆tx, ∆ty)/mm
	Coordinates of the Actual Treatment Center (x, y)/Pixels	The Registration-Derived Positioning Error (tx, ty)/mm		
Group 1	(128.5, 126.5)	(0, 0)	(0, 0)	(0, 0)
Group 2	(133.5, 126.5)	(−10, 0)	(−10, 0)	(0, 0)
Group 3	(128.5, 128.5)	(0, −4)	(0, −3)	(0, −1)
Group 4	(125.5, 122.5)	(6, 10)	(5, 10)	(1, 0)
Group 5	(127.5, 128.5)	(2, −4)	(3, −3)	(−1, −1)

## Data Availability

The data presented in this study are available in this article. Further inquiries can be directed to the corresponding author.
